# Dietary Behavior: An Interdisciplinary Conceptual Analysis and Taxonomy

**DOI:** 10.3389/fpsyg.2018.01689

**Published:** 2018-09-20

**Authors:** F. Marijn Stok, Britta Renner, Julia Allan, Heiner Boeing, Regina Ensenauer, Sylvie Issanchou, Eva Kiesswetter, Nanna Lien, Mario Mazzocchi, Pablo Monsivais, Marta Stelmach-Mardas, Dorothee Volkert, Stefan Hoffmann

**Affiliations:** ^1^Department of Psychology, University of Konstanz, Konstanz, Germany; ^2^Health Psychology, The Institute of Applied Health Sciences, University of Aberdeen, Aberdeen, United Kingdom; ^3^Department of Epidemiology, German Institute of Human Nutrition Potsdam-Rehbruecke, Nuthetal, Germany; ^4^Experimental Pediatrics and Metabolism, University Children’s Hospital, Heinrich Heine University Düsseldorf, Düsseldorf, Germany; ^5^Centre des Sciences du Goût et de l’Alimentation, AgroSup Dijon, CNRS, INRA, Université Bourgogne Franche-Comté, Dijon, France; ^6^Institute for Biomedicine of Aging, Friedrich-Alexander-Universität Erlangen-Nürnberg, Erlangen, Germany; ^7^Department of Nutrition, Faculty of Medicine, University of Oslo, Oslo, Norway; ^8^Department of Statistical Sciences, University of Bologna, Bologna, Italy; ^9^Centre for Diet and Activity Research, MRC Epidemiology Unit, Institute of Metabolic Science, University of Cambridge, Cambridge, United Kingdom; ^10^Department of Biophysics, Poznan University of Medical Sciences, Poznań, Poland; ^11^Department of Marketing, Institute of Business Administration, Kiel University, Kiel, Germany

**Keywords:** taxonomy, ontology, cumulative science, dietary intake, diet, food choice, nutrition, eating behavior

## Abstract

**Background:** Dietary behavior encompasses many aspects, terms for which are used inconsistently across different disciplines and research traditions. This hampers communication and comparison across disciplines and impedes the development of a cumulative science. We describe the conceptual analysis of the fuzzy umbrella concept “dietary behavior” and present the development of an interdisciplinary taxonomy of dietary behavior.

**Methods:** A four-phase multi-method approach was employed. Input was provided by 76 scholars involved in an international research project focusing on the determinants of dietary behavior. Input was collected from the scholars via an online mind mapping procedure. After structuring, condensing, and categorizing this input into a compact taxonomy, the result was presented to all scholars, discussed extensively, and adapted. A second revision round was then conducted among a core working group.

**Results:** A total of 145 distinct entries were made in the original mind mapping procedure. The subsequent steps allowed us to reduce and condense the taxonomy into a final product consisting of 34 terms organized into three main categories: Food Choice, Eating Behavior, and Dietary Intake/Nutrition. In a live discussion session attended by 50 of the scholars involved in the development of the taxonomy, it was judged to adequately reflect their input and to be a valid and useful starting point for interdisciplinary understanding and collaboration.

**Conclusion:** The current taxonomy can be used as a tool to facilitate understanding and cooperation between different disciplines investigating dietary behavior, which may contribute to a more successful approach to tackling the complex public health challenges faced by the field. The taxonomy need not be viewed as a final product, but can continue to grow in depth and width as additional experts provide their input.

## Background

Malnutrition (including over- and under-nutrition as well as nutritional deficiencies) and the non-communicable diseases related to it constitute one of the largest challenges that public health is currently facing (e.g., Kelly et al., 2008; Popkin, 2011; Agarwal et al., 2013). As of yet, attempts to address unhealthy diets and curb unhealthy diet-related practices have had limited success (e.g., Snyder, 2007; Traill et al., 2010; Capacci et al., 2012). In order to design effective policies and interventions, it is crucial to gain insight into the complex multitude of factors that shape and influence dietary behavior (see also Stok et al., 2017). As research into these determinants is scattered across many disciplines ranging from nutritional epidemiology to psychology and from anthropology to economics, combining the existing evidence into one coherent overview requires effective synthesis of the existing literature across a multitude of disciplines. Due to the widely varying research traditions across and even within these various disciplines, the ability to successfully synthesize the evidence hinges on a clear definition and shared understanding of the exact dietary behavior *outcomes* that are assessed, so that it is possible to establish exactly which determinants are related to which aspects of diet and which aspects of diet change in response to which determinant-aimed intervention.

Crucially, however, “dietary behavior” and related terms such as “diet”, “nutrition”, “dietary intake”, “eating behavior”, “eating habits”, and “food choice” are fuzzy umbrella concepts, which are used inconsistently across different disciplines and research traditions, and which often include a multitude of qualitatively different outcomes lumped together. These outcomes cover all aspects related to dietary behavior from foraging to ingestion and range from the intake of single nutrients to patterns of entire diets, from disordered eating to eating habits, from food preferences to food preparation. Moreover, while standardized classifications of certain aspects of dietary behavior exist within single disciplines [e.g., a classification of nutrients within dietetics and nutrition science (King et al., 2007) and a standardized method for classifying assessment of diet within epidemiology (Lachat et al., 2016)], no consistent terminology that is shared across disciplines is currently available for these concepts. As a consequence, the same term can be used to represent different things across disciplines and researchers and conversely, one concept might be referred to with various different terms (see also Larsen et al., 2017). For example, some researchers have used the term “dietary intake” to refer to intake of specific food groups, such as sugar-sweetened beverages (Ievers-Landis et al., 2016) or fruits, vegetables, and unhealthy snacks (Tăut et al., 2015), while others have used the same term to refer to the nutritional composition (e.g., Moore-Schiltz et al., 2015) or the total energy content (e.g., Lombard et al., 2015) of people’s diet. Even within one discipline the same terms may be used differently.

### Toward a Standardized Taxonomy

This complexity of dietary behaviors, paired with a lack of terminological clarity, consistency and consensus has hindered the ability to compare findings about relevant determinants from the different disciplines. This, in turn, hampers our understanding of the determinants of dietary behavior and impedes the development of a cumulative science. We posit that a more harmonized terminology for the multitude of concepts related to dietary behavior is a prerequisite for the advancement of both research and practice. To this end, we conducted a thorough conceptual analysis in an interdisciplinary working group and subsequently developed a taxonomy of human dietary behavior across the lifespan that can be used across disciplines and that harmonizes and categorizes the various concepts typically considered under the broad umbrella of “dietary behavior”.

A standardized taxonomy of dietary behaviors offers several advantages to research. By providing systematic assessment and categorization, a shared taxonomy illuminates and makes explicit the large extent of diversity that exists within this topic, and allows for standardized application of definitions. As such, a shared taxonomy will encourage mutual understanding about both the different aspects of diet and dietary behavior, as well about the specific determinants affecting each of these different aspects. In addition, knowledge exchange and sharing of determinant information between countries and across disciplines becomes substantially simpler when all disciplines can refer to one standardized taxonomy, and potential misunderstandings will become easier to resolve. Extending this argument further, a shared taxonomy can also expedite the pooling of results from research across countries and disciplines. Data aggregation allows for the conduction of more powerful analyses, like secondary data analysis (Vartanian, 2010) on pooled data (e.g., Jakobsen et al., 2009) or federated meta-analysis (a procedure which allows for data analysis across multiple cohorts without necessitating direct access to the individual data, see, e.g., Doiron et al., 2013), and help build a more reliable and powerful evidence base. Data pooling can shed new light on potential drivers of different aspects of dietary behavior that cannot be achieved with single data sets within single disciplines, thus facilitating our ability to design effective determinant-aimed policies and interventions that can be targeted at specific aspects of dietary behavior.

Our aim of standardizing terminology to facilitate interdisciplinary collaboration, knowledge sharing, and data pooling coincides with a general development within overall scientific practice. For example, a recent development involves the creation of ontologies [a method for systematically assessing and registering the properties of concepts or constructs within a certain domain, as well as the interrelations between these phenomena (Groß et al., 2016; Larsen and Bong, 2016; Larsen et al., 2017)] to facilitate knowledge accumulation, synthesis and integration. Ontologies have been developed both within nutrition-related research [e.g., the Ontology for Nutritional Studies from the ENPADASI project (Pinart et al., 2018) and the development of the standardized STROBE-nut statement aimed at strengthening reporting of nutritional epidemiological studies (Lachat et al., 2016)] as well as in other scientific domains (e.g., Cimino and Zhu, 2006; The Gene Ontology Consortium, 2017).

### Current Study

The current article describes the conceptual analysis of the fuzzy umbrella concept “dietary behavior” and related concepts conducted within an interdisciplinary, international workgroup of researchers that was formed in the context of the European research network and knowledge hub Determinants of Diet and Physical Activity (DEDIPAC; see Lakerveld et al., 2014), and the congruent development of a taxonomy of dietary behavior. This workgroup was, amongst other tasks, in charge of the development of a framework of the determinants of dietary behavior across the lifespan (Stok et al., 2017). Conceptually analyzing, inventorying, harmonizing, and categorizing the multitude of concepts that can be considered to fall under the broad “dietary behavior” term was considered a necessary precursor to the development of such a framework of determinants, and this has resulted in the taxonomy presented in the current paper. It is important to note that, for the purposes of the DEDIPAC project, dietary behavior was viewed as the *outcome* of its determining factors. It should be noted that, of course, from the perspective of more upstream health consequences (such as type 2 diabetes, obesity, or heart disease), dietary behavior in turn would be considered as the cause or determinant. For the purposes of the DEDIPAC project, however, dietary behavior was thus considered as the *outcome* of certain determinants, and the framework that is presented here was conceptualized as such.

## Methods

### Workgroup

The workgroup consisted of 76 scholars with varying academic backgrounds, ranging from biology to economics and from nutrition to anthropology, and from eleven different countries (see **Table 1** for more details). What all members shared was a research focus on diet and/or diet-related aspects, such as nutrition-related non-communicable diseases. The workgroup was managed and led by two of the authors (FMS and BR), and followed previous examples of successful multidisciplinary academic cooperation in the field of obesity-related behaviors (e.g., Booth et al., 2001). A core workgroup, consisting of representatives of different scientific disciplines (FMS, BR, HB, RE, EK, MS-M, DV, and SH) was also created to facilitate efficient discussion. The core workgroup communicated at least every two weeks throughout the project period. The core workgroup members in turn each guided, and maintained contact with, a subgroup of the remaining workgroup members throughout the project period.

**Table 1:** Scientific disciplines and countries represented within the working group.

**Scientific disciplines**	Anthropology
	Biology/Human Biology
	Dietetics
	Economics
	Epidemiology
	Food Engineering
	Food Science
	Food Technology
	Geriatrics
	Health Promotion
	Marketing and Consumer Research
	Medicine
	Nutrition Science
	Pediatrics
	Physical Education
	Physiology
	Physiotherapy
	Psychiatry
	Psychology
	Public Health
	Social Demography
	Sports Sciences
	Statistics

**Countries**	Belgium
	Finland
	France
	Germany
	Ireland
	Italy
	Netherlands
	Norway
	Poland
	Spain
	United Kingdom

### Working Process

**FIGURE 1:**
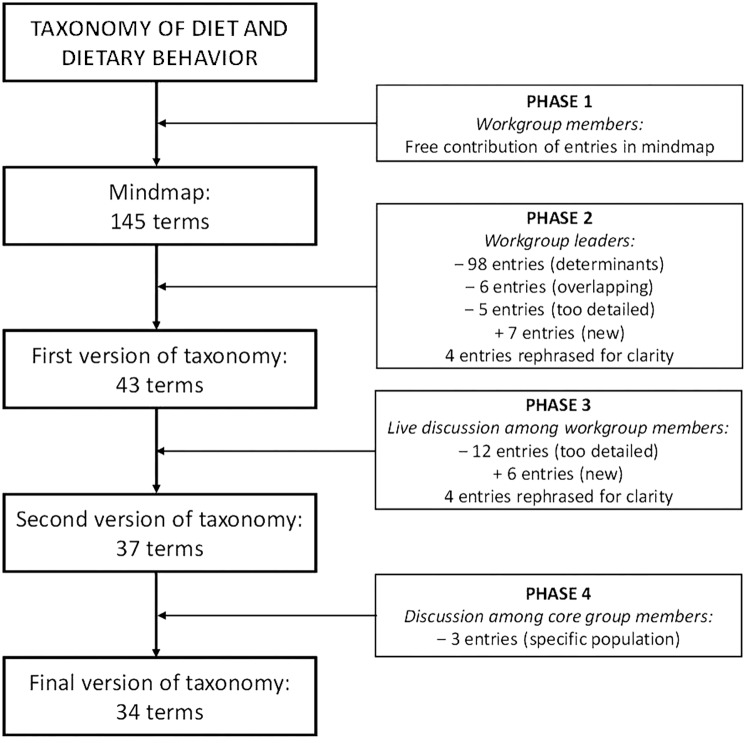
Flowchart of the working process and development of the taxonomy throughout the four phases.

The conceptual analysis and taxonomy development were conducted using a four-phase multi-method approach. The first, second, and fourth phases occurred de-centralized (that is, each contributor or group of contributors worked independently), while the third phase was centralized, with the workgroup members assembled at one location at one specific time. A flowchart (see **Figure 1**) graphically depicts the working process and the development of the taxonomy throughout the different phases. The ethics committee of the University of Konstanz decided that this project falls outside the range of projects requiring an IRB statement (decision IRB17KN9_001).

### Phase 1 – Conceptual Analysis

In the first phase, an online mind mapping (Wexler, 2001) tool called MindMeister^1^ was used to analyze the different interpretations and meanings of the fuzzy umbrella concept “dietary behavior”. Workgroup members were instructed to nominate all specific outcomes that they felt fall within this broad umbrella concept. The term “dietary behavior” was used as the starting point because this was also the term used in the DEDIPAC research proposal. However, workgroup members were instructed to consider this broadly, and also take into account related umbrella concepts such as “nutrition”, “food choice”, *et cetera*. The technique of online mind mapping was chosen because it is well-suited for the comprehensive, structured and visually accessible collection of input from different people simultaneously or in rapid succession, while keeping track of which input was provided by whom. In the online mind mapping tool, work group members added separate text boxes for each individual outcome they considered relevant. The tool allowed for the specification of both a hierarchical structure between different text boxes (with higher-order text boxes indicating general, more encompassing outcomes and lower-order text boxes indicating underlying, more detailed outcomes), as well as a categorical structure between different text boxes (with text boxes in the same categorical branch of the structure indicating qualitatively similar outcomes). It was also possible to indicate relations (by means of arrows) between text boxes that are not otherwise connected in the hierarchical or categorical structure. While contributing their ideas, workgroup members were thus automatically required to also consider the categorical and hierarchical structure of the concepts they listed. Communication was possible within the program itself via comments, such that different workgroup members could discuss and resolve areas of disagreement in real time.

Several guidelines for the nomination phase were provided by the workgroup leaders. First, each partner was given a unique color and symbol in the program, so that it was possible to keep track of partners’ contributions. Partners were encouraged to also indicate their name when providing input, to ensure that everybody knew who contributed which input, from which scientific background, facilitating effective discussion about potential areas of disagreement. Second, partners were informed that nomination of outcomes was possible both based on scientific evidence (e.g., literature review) as well as on expert knowledge and experience (e.g., previous use in own research), in order to arrive at the most complete and comprehensive mind map possible. Third, partners were asked never to delete anything from the mind map, but to communicate disagreement with existing input via comments instead. The mind map was made available to all partners involved in the workgroup to review and edit for one month, after which the final mind map was downloaded and stored.

### Phase 2 – Structuring of Dietary Terminology

In phase 2, the outcomes entered in the mind map were reduced, combined, and categorized into a compact taxonomy by the workgroup leaders (BR and FMS). All decisions made were recorded and discussed with the larger working group in a later live discussion session (Phase 3, see below). The workgroup leaders first merged entries that were clearly referring to the same outcome. In case there was any doubt about whether two entries referred to the exact same outcome, both were retained, to be further discussed in a later phase. Second, the workgroup leaders determined whether each entry indeed specified a distinct diet-related outcome. Entries that did not meet this criterion were not retained (e.g., those which specified dietary determinants rather than true diet-related outcomes). The remaining valid and unique entries were then scrutinized with regard to content and position in the categorical structure. Entries were structured systematically, grouping conceptually related outcomes together. In Phase 1, workgroup members disagreed on multiple issues (e.g., placement of entries in the taxonomy, relevance of certain entries). In these cases, the workgroup leaders made decisions based on majority opinion (which could be deduced from the accompanying comments in the mind map program) and, whenever possible, checked these decisions against the available evidence in the literature. When no clear majority opinion or best evidence-based option was evident, the issue was noted down to be put forward to further discussion in the next phase. The entries in the resulting categorization of outcomes were then scrutinized more closely for level of detail (position in the hierarchical structure). The decision was made not to include outcomes related to individual food items or nutrients, in order to keep the taxonomy compact, condensed, and consistent with our focus on research targeting overall human dietary behavior.

### Phase 3 – Live Discussion

In the third phase, the reduced and condensed taxonomy was presented to all workgroup members during a live DEDIPAC project meeting. The taxonomy was displayed on a large screen and suggested changes to the taxonomy were processed on-screen in real time, so that workgroup members could see the effects of each potential change and evaluate its desirability. In a first discussion round, the categorization issues remaining from the previous phase were discussed by the moderators (FMS and BR) and resolved. In a second discussion round, workgroup members were able to provide any remaining comments or suggestions, which were then discussed amongst all members until resolved. All occurring disagreements could be resolved through mutual discussion, such that the final product was agreed upon. The end product of this phase was a further reduced, “pre-final” taxonomy approved by all of the workgroup members who were present.

### Phase 4 – Final Coordination

In the fourth phase, the “pre-final” taxonomy was thoroughly discussed once more by the core workgroup and, where necessary, edited. Finally, the workgroup leaders compared the final taxonomy to the original mind map, so as to ensure that the input provided by the workgroup members in phase 1 was correctly and exhaustively represented in the taxonomy. After concluding that this was the case, the resulting taxonomy was finalized as the end product of the work process. The terms in the final taxonomy were then defined by the core workgroup. Definitions were based on the working definitions of the terms used within the entire working group during the project period, and checked by the workgroup leaders against the commonly accepted terms in the literature.

## Results

### Phase 1 – Conceptual Analysis

A total of 43 unique contributors provided input on the mind map. Unique contributors in some cases collected and summarized inputs from multiple colleagues in their research group, meaning that the mind map contains input of substantially more than 43 workgroup members. The final mind map (available from the authors) consisted of a total of 145 entries that all represented, according to the members of the workgroup, different diet-related outcomes. The entries differed to a large extent with regard to their level of detail; both high-order (e.g., “intake of healthy foods”) as well as much more specific, lower-order (e.g., “fish oil intake”) outcomes were mentioned. Importantly, the mind map process organically gave rise to a logical and concise main categorization of the terms: the workgroup members grouped most of the text boxes into three main parts, which at the end of the entry period were found to represent three meaningfully different categories. These three categories (food choice, eating behavior, and dietary intake/nutrition) will be further described in the section “final taxonomy”. Some entries were not included in one of these three main parts of the mind map, but rather entered as isolated terms.

### Phase 2 – Structuring of Dietary Terminology

In the second phase, six entries were removed because they overlapped (e.g., meal content and meal composition). Furthermore, as the ultimate target of the working group was the development of a framework of determinants, several members misinterpreted the purpose of the mind map, and thought that the purpose was to collect both dietary behavior-related outcomes as well as potential determinants of these outcomes. A total of 98 entries specified determinants of dietary behavior (e.g., “social context” and “boredom”) rather than dietary behavior-related outcomes themselves, and were removed again for that reason [determinant collection occurred at a later stage during the working process and is described elsewhere (Stok et al., 2017)]. In some cases, it was not entirely clear whether an entry was a determinant or outcome (e.g., “where one eats” and “when one eats”) – members expressed different opinions about these entries. The workgroup leaders made decisions in these cases based on majority opinion and, whenever possible, evidence from the relevant literature. When no clear majority opinion or best evidence-based option was evident, the issue was noted down to be further discussed in the next phase. Finally, five entries were removed because they were overly specific for a general taxonomy (e.g., olive oil and particular vitamins). The remaining 36 entries were rephrased when necessary (*n* = 4, e.g., “diet diversity” into “diversity of dietary pattern”). In addition, the workgroup leaders added seven entries (e.g., “dieting”) which they felt were missing from the mind map, leading to a total of 43 entries. All of these decisions were recorded and extensively discussed with the entire working group during the live discussion session (Phase 3).

Using the three-legged structure of the original mind map as a guideline, the 43 entries were organized into a concise taxonomy with a maximum of four hierarchical levels. Placement of the entries within the taxonomy was based on the suggestions by the workgroup members in the online mind map. Comments in the online mind map program showed that members disagreed about the best placement within the taxonomy in several cases (e.g., whether “intentions” should be placed under the “food choice” leg or under the “eating behavior” leg, and whether “healthy intake” and “unhealthy intake” should be separate entries within the “dietary intake/nutrition” leg, or whether these should be organized hierarchically as sub-entries of “dietary pattern”). Again, in these cases the workgroup leaders made decisions based on majority opinion and evidence from the relevant literature; when this was not possible the issue was noted down to be further discussed in the next phase.

### Phase 3 – Live Discussion

This 43-entry taxonomy was used as the starting point for the discussion during the live meeting. About 50 workgroup members were present at the live meeting. The process of reducing, combining and categorizing the terms of the mind map by the workgroup leaders was presented and extensively discussed. Areas of disagreement that the workgroup leaders noted in Phase 2 were given extensive attention in the live session and discussed until they could be resolved through consensus. The live discussion session resulted in the removal of further 12 entries because the workgroup members considered these entries too detailed (e.g., “snacks”, “soft-drinks” and “Mediterranean diet”). The workgroup members further agreed on adding six other entries (e.g., “healthiness of dietary pattern”) which they considered to be missing from the previous version, and to rephrase four more of the original entries (e.g., “healthy vs. unhealthy choices” into “preferences”). Beyond these alterations, the taxonomy drawn up by the workgroup leaders was approved by the working group. The partners also agreed upon the main three-category structure (food choice, eating behavior, and dietary intake/nutrition) that had already emerged from their preliminary organization in the original mind map. Together, these changes meant that the second version of the taxonomy consisted of 37 entries. Importantly, all workgroup members present at the live meeting (stemming from diverse research disciplines and from various countries) confirmed that the outcomes they typically focused on in their line of work were adequately represented in the taxonomy.

### Phase 4 – Final Coordination

**FIGURE 2:**
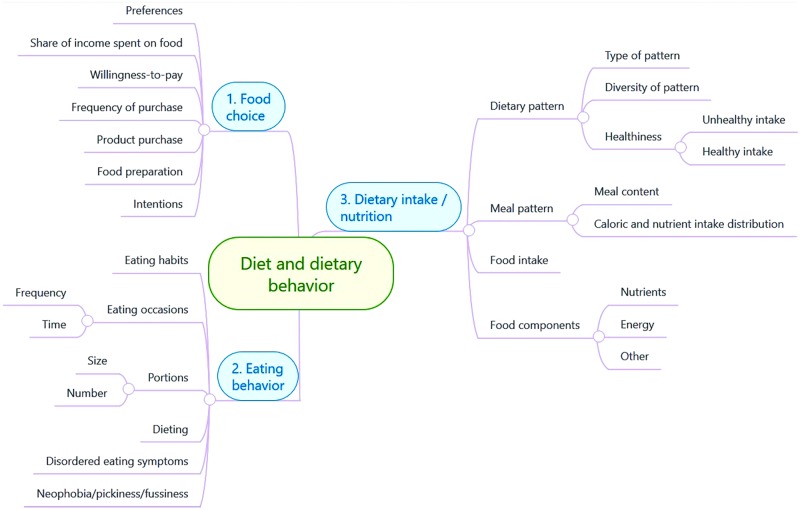
The taxonomy of outcomes related to dietary behavior; figure prepared using MindMeister.com.

A thorough discussion in the core workgroup led to the decision to remove three lower-level entries that were relevant only to a very specific target population (namely newborns; all three entries were related to breastfeeding), so as to ensure that all terms in the taxonomy were applicable to the entire lifespan. This was discussed with and approved by the workgroup members who had originally included these entries in the mind map. This resulted in a taxonomy including 34 entries. The core workgroup also rearranged the lower-level order of some terms within the three main categories to arrive at the most logical representation; this did not change the content of the taxonomy in any way. The core workgroup members discussed this change within the subgroups to ensure that all workgroup members agreed with this change. Finally, the workgroup leaders compared the resulting taxonomy with the original input by the workgroup members and were able to conclude that the taxonomy represented the workgroup members’ original input. The process was therefore considered to be successfully concluded and the taxonomy was finalized. The final taxonomy is depicted in **Figure 2**. A table of definitions of the terms in the final taxonomy was then created (see **Table 2**), based on the working definitions of the terms used within the entire working group during the project period. The workgroup leaders checked these working definitions against the commonly accepted terms in the literature.

**Table 2:** Definitions of the terms in the taxonomy.

Term					Definition (sample reference provided for all terms except the four umbrella terms)
*DIET AND DIETARY BEHAVIOR*					Overall umbrella term referring to all phenomena related to food choice, eating behavior, and dietary intake/nutrition.
1	*Food choice*				Umbrella term for behaviors and other factors occurring before food actually reaches the mouth.
2		Preferences			The extent to which food items are considered desirable or undesirable including underlying preferences (attitudes toward, e.g., taste, odor, color, or quality and health-related concerns) and expressed preferences (actual choice of one food item over another) (Rozin, 2006; Bernheim and Rangel, 2009).
3		Share of income spent on food			The share of income (of one person, family, or household) that is spent on the totality of food one consumes (Clements and Si, 2017).
4		Willingness-to-pay			Largest sum of money one is willing to pay for a certain food product (Varian, 1992).
5		Frequency of purchase			How often a certain food product is purchased in a specific time frame (Kim and Rossi, 1994).
6		Product of purchase			Which food product is purchased (Mohd Suki, 2016).
7		Food preparation			Factors associated with how food is prepared for consumption (Larson et al., 2006).
8		Intentions			What one intends or plans to choose, buy, or consume (Conner et al., 2002).
9	*Eating behavior*				Umbrella term for outcomes related to the actual act of consumption.
10		Eating habits			The typical/habitual eating behaviors that one has developed over time, often triggered automatically in response to contextual cues that have been associated with their performance (Gardner, 2015).
11		Eating occasions			The actual eating event; may include also the physical and social context in which one eats (e.g., how often and when one eats at home; which portion size or number one eats when being with others) (Leech et al., 2015a).
12			Frequency		How often one eats (Gomez et al., 2015).
13			Time		When one eats (which moment of the day) (Kupek et al., 2016).
14		Portions			The amount one eats (Amougou et al., 2016).
15			Size		A specification of the amount one eats in terms of the size of a standard portion(Nielsen and Popkin, 2003).
16			Number		A specification of the amount one eats in terms of the amount of standard portions (Benton, 2015).
17		Dieting			To eat sparingly (typically in order to lose weight or maintain a certain body weight) (Heatherton and Polivy, 1990).
18		Disordered eating symptoms			Signals of abnormal eating (e.g., anorexia and binge eating) (Rohde et al., 2015).
19		Neophobia/pickiness/fussiness			Different aspects of selective eating (Dovey et al., 2008).
20	*Dietary intake/nutrition*				Umbrella term for outcomes that break down the content of what exactly is being consumed.
21		Dietary pattern			Specific combination of foods and beverages one eats on a regular basis, which includes a specific mix of nutrients (Hu, 2002).
22			Type of pattern		Characterization of the dietary pattern, e.g., in Western, Mediterranean, and Prudent (Hu, 2002).
23			Diversity of pattern		Diversity of foods and beverages consumed (within a specific pattern) (Hu, 2002).
24			Healthiness		Extent to which the (combinations of) foods and beverages consumed are considered to have a negative or positive effect on one’s health (Ocké, 2013).
25				Unhealthy intake	Consumption of (combinations) of foods and beverages attributed to have a negative effect on one’s health, e.g., sugar sweetened beverages (Ocké, 2013).
26				Healthy intake	Consumption of (combinations) of foods and beverages attributed to have a positive effect on one’s health, e.g., fruits and vegetables (Ocké, 2013).
27		Meal pattern			Frequency, time and content of meals one eats throughout the day as well as distribution of energy and nutrient intake across the meals (Leech et al., 2015b).
28			Meal content		Combination of food items within a meal (Leech et al., 2015b).
29			Caloric and nutrient intake distribution		Distribution of (daily) energy and nutrient intake across the main meals and snacks (Leech et al., 2015b).
30		Food intake			Amount/servings of food items one eats within a specific time frame (mostly per day) (Illner et al., 2010).
31		Food components			Food items or ingredients of a mix food that contain certain amounts of energy, nutrients, and non-nutritive substances (Coultate, 2009).
32			Nutrients		The nutrients (carbohydrates, protein, fat, vitamins, minerals, and water) in a food item (Coultate, 2009).
33			Energy		The caloric content of a food item (Coultate, 2009).
34			Other		Non-nutritive food components of a food item (Coultate, 2009).

### Final Taxonomy

The three main categories in the final taxonomy represent wholly different aspects of diet and dietary behavior. Taken together, these three categories are a concise yet holistic representation of the range outcomes under the umbrella concept of “dietary behavior”, including outcomes relating to the beginning of the eating process (long preceding the actual act of ingesting food), all the way through to terms regarding constituents of the food that has been ingested. Broadly put, the first main category, named *Food Choice*, represents behaviors and other factors occurring before food actually reaches the mouth. This category is further broken down into seven distinct outcomes: *preferences*, *share of income spent on food*, *willingness-to-pay*, *frequency of purchase*, *product purchase*, *food preparation*, and *intentions*. The second main category, *Eating Behavior*, encompasses all the outcomes related to the actual act of consumption. This category is further broken down into six distinct outcomes (some of which are broken down further, see **Figure 2**): *eating habits*, *eating occasions*, *portions*, *dieting*, *disordered eating symptoms*, and *neophobia/pickiness/fussiness*. Finally, the third main category, *Dietary Intake/Nutrition*, includes all outcomes that break down the content of what exactly is being consumed. This category consists of four main outcomes (some of which are further divided into more detailed outcomes, see **Figure 2**): *dietary pattern*, *meal pattern*, *food intake*, and *food components*.

## Discussion

A conceptual analysis of the fuzzy umbrella concept “dietary behavior” was carried out in an interdisciplinary, international workgroup of researchers, and resulted in the development of a taxonomy of diet-related outcomes. The taxonomy was developed with the aims of demonstrating the breadth of outcomes grouped under the broad umbrella concept “dietary behavior”, and categorizing these outcomes in a concise consensus structure. Having such a taxonomy in place can help interdisciplinary understanding and knowledge exchange, thereby facilitating cross-disciplinary research. Starting from a mind mapping approach and ending in a live consensus meeting, diet-related outcomes were grouped into three main categories: food choice, eating behavior, and dietary intake/nutrition. Each category was further broken down into more specific outcomes, with the final taxonomy consisting of 34 terms (see **Figure 2**).

### Prospective Use of the Taxonomy

Evidence from other health-related research fields suggests that the development and application of consensus taxonomies can catalyze research by lowering the threshold for knowledge exchange, data sharing and data pooling efforts, all of which is urgently required in order to more successfully face the complex, multifaceted problems challenging public health. For example in the (bio)medical field, the important benefits of developing and employing standardized taxonomies (e.g. of diseases, mental disorders and genes) has been advocated for decades and remains an important agenda topic (e.g., Sartorius, 1974; World Health Organization, 1974; National Research Council [US], 2011; The Gene Ontology Consortium, 2017). In the field of geriatrics, the development of a taxonomy of outcomes enabled major advances in the field of fall prevention, through facilitating alignment and comparison of earlier research and the pooling of data (Lamb et al., 2005; Hauer et al., 2006). Researchers in the field of sedentary behavior (which, like malnutrition, is an important challenge currently facing public health) have also recently established a taxonomy to further the field (Chastin et al., 2013).

Importantly, the current taxonomy complements similar initiatives within the fields of nutrition and behavior change. For example, in a recent publication describing the advantages of using standardized ontologies for behavior change interventions (Larsen et al., 2017), the authors indicated that one of the key components necessary for a behavior change ontology would be a classification of “behavioral outcomes”. The currently proposed taxonomy can be useful in fulfilling this requirement, as it categorizes and standardizes diet-related outcomes. Moreover, recent efforts to promote standardization of the description of dietary *assessment* in empirical research (Lachat et al., 2016) combines well with the efforts presented in this article to more accurately distinguish between the different *constructs* included in the umbrella term dietary intake and nutrition (the second leg of the taxonomy). By combining a standardized inventory of the different aspects of dietary behavior with a standardized inventory of the ways to assess these aspects, empirical research in the area of nutrition can be more easily compared and pooled. Arguably the most extensive effort to date in the field of nutrition stems from the ENPADASI research project (Pinart et al., 2018), which aims to develop an all-encompassing ontology of all aspects related to nutrition (food components, foods, the diet, the individual, the health, and the diseases). The ontology currently comprises of tens of thousands of entries describing different constructs and the relations between them. While this ontology is thus extremely extensive and highly useful for standardizing results from nutritional epidemiological studies, its extensiveness and complexity makes it less fit for day-to-day research collaboration and referral between researchers – the currently proposed taxonomy being a more manageable tool for such purposes. In order to maximize the collective potential of all of these initiatives, the creation of a “meta-ontology”, unifying these different initiatives, would be a highly useful step forward.

The taxonomy can be helpful both in day-to-day collaborations between scientists from different disciplines, as well as in more formal efforts to compare and synthesize earlier research results from different disciplines. As an example of the former, the taxonomy has facilitated understanding between the DEDIPAC workgroup members, ensuring a common representation of outcomes related to dietary behavior throughout the project. To illustrate, two or more researchers in the working group would frequently state that they each investigated a certain outcome, for example “eating behavior”, only to later conclude that the exact outcomes they employed were in very different locations of the taxonomy (for example, “eating occasions” versus “healthy intake”). By referencing the taxonomy and determining one’s figurative “location” on the taxonomy, mutual understanding was enhanced. The potential benefits of the taxonomy extend beyond the scope of this single research project, however, to the general field of diet-related research and the substantial challenge of diet-related health problems faced in public health (such as the obesity epidemic). As an example of manner in which the taxonomy can facilitate efforts to compare and synthesize research results, we come back to the issues (presented in the introduction) of one and the same term being used to represent different things across disciplines and researchers and conversely, of one concept being referred to with various different terms (called the “jingle fallacy” and “jangle fallacy”, respectively, Larsen and Bong, 2016). This can lead to substantial problems: studies may not actually be as comparable as one thinks (in the former case) or relevant studies describing the same thing may be missed because they are called differently (in the latter case). By referencing the taxonomy and organizing empirical evidence according to the categories of the taxonomy, such problems can be avoided. Finally, in the context of determinant research, the taxonomy can help researchers specify which exact aspects of dietary behavior certain determinants are related to. Many given determinants, namely, are not predictive of simply any aspect of dietary behavior, but rather are related only to certain specific outcomes (or at least have only been proven to be related to these specific outcomes). As an example, consider the determinant of social norms, which has been shown to be strongly related to such outcomes as healthy and unhealthy intake, and eating behavior, but which has not been researched in relation to an outcome like food components. Conversely, there is substantial evidence indicating that seasonality affects outcomes like dietary pattern and nutrient intake, but this determinant is not typically considered in relation to such outcomes like willingness-to-pay and dieting.

With the publication of the taxonomy, our aim is to open it up to the field at large. The currently presented conceptual analysis and taxonomy should not be considered as a finite end-product, but rather can be considered as a dynamic starting point to which the field can continue to add content, potentially expanding the taxonomy both in width and depth. To that end, the taxonomy has been made publicly available together with the DONE framework^2^^,^^3^. A core group of people involved in the creation of the taxonomy will maintain and update the website on which it is available. Using a standard form, visitors can provide suggestions for improvement (e.g., changes or additions). These suggestions will be appraised for validity and plausibility by the core working group, and the taxonomy can be updated in an event-based manner. Visitors can also provide general feedback on the taxonomy. The taxonomy is also disseminated through presentation at scientific conferences (e.g., Stok and Renner, 2015a,b). Use of the taxonomy by the scientific community will be monitored and recorded by the core working group, with usage data being presented on the website.

### Limitations

While the current conceptual analysis and taxonomy development were conducted within a substantial, interdisciplinary and international working group as described above, this group nevertheless represents only a limited selection of people working in the field of dietary behavior. In order for the taxonomy to reach its potential as a useful tool for cross-disciplinary collaboration, it should be commonly adopted across disciplines. That means, firstly, that there must be a general consensus on the relevance of having a taxonomy of dietary behavior and, secondly, that there should be a widespread agreement on the content and structure of the taxonomy. The taxonomy will be disseminated to the field through various traditional channels (research articles, conference presentations) but, importantly, is also available online on an openly accessible website (see footnote 3), which also hosts the framework of determinants of dietary behavior developed in conjunction with this taxonomy within the DEDIPAC project. This website also provides the possibility for discussion and comments where experts can suggest additions and changes to the DONE framework and the taxonomy.

Somewhat related to the previous issue, the working group was an exclusively European group, and also predominantly a north-western European group, and its findings should be placed in this context. Nevertheless, the working group included researchers from a wide range of European countries. These countries differ substantially and, crucially, predictably, with respect to socio-cultural, socio-economic, and socio-demographic characteristics. Comparing findings from the different countries could provide some indications as to how results might be extrapolated to other Western countries that were not part of the working group, though of course such extrapolation should be further examined and empirically tested for validity. Moreover, our findings cannot be readily applied to non-Western cultures as such countries were not part of the current working group.

Another limitation is the fact that the taxonomy was developed (a) with a specific purpose in mind; ultimately, the taxonomy was developed to facilitate research on the *determinants* of dietary behavior, and (b) to predominantly cover dietary behavior outcomes applicable to the general population, across the entire lifespan. The taxonomy may therefore need to be adjusted and expanded in order to meet also the needs of researchers interested in the more upstream pathway of dietary behavior as the exposure factor for various health consequences. Moreover, similar expansions in both depth and width may be necessary to accommodate also more detailed dietary behaviors relevant for specific populations (e.g., newborns, elderly, people who require artificial nutrition, *et cetera*). Crucially, because the taxonomy is not considered finite but rather is suggested as a starting point that can be improved upon, such changes can indeed be realized when deemed necessary or useful by other researchers in the field.

## Conclusion

Within a large group of researchers, from different European countries and with varying academic backgrounds, we conducted a conceptual analysis of the fuzzy umbrella concept “dietary behavior”, and developed a taxonomy of outcomes attributed to this fuzzy concept. This taxonomy demonstrates the extent of variation that exists within the field of diet, and succeeds in categorizing this variation into a concise structure. We propose that the taxonomy can be put to good use in promoting understanding, knowledge exchange, and data sharing between disciplines and countries, all of which are crucial components of a new, more holistic, and more successful approach to tackling the complex challenges faced by the field.

## Author Contributions

BR and FMS conceptualized the analysis and development of the taxonomy, devised the methodology, and drafted the manuscript. FMS created the first version of the taxonomy. BR, JA, HB, RE, SI, EK, NL, MM, PM, MS-M, DV, and SH contributed to the revision of the taxonomy. JA, HB, RE, SI, EK, NL, MM, PM, MS-M, DV, and SH critically reviewed and edited the manuscript. All authors were involved in the mind mapping procedure and approved submission of the manuscript in its current form.

## Conflict of Interest Statement

The authors declare that the research was conducted in the absence of any commercial or financial relationships that could be construed as a potential conflict of interest.The reviewer CL and handling Editor declared their shared affiliation.

## Footnotes

1 www.mindmeister.com

2 www.uni-konstanz.de/DONE

3 www.uni-konstanz.de/DONE/taxonomy
